# PF-06409577 Activates AMPK Signaling and Inhibits Osteosarcoma Cell Growth

**DOI:** 10.3389/fonc.2021.659181

**Published:** 2021-07-14

**Authors:** Yun-Rong Zhu, Xiang-Yang Zhang, Qiu-Ping Wu, Cheng-Jian Yu, Yuan-Yuan Liu, Yun-Qing Zhang

**Affiliations:** ^1^ Department of Orthopedics, Affiliated Jiangyin Hospital of Medical College of Southeast University, Jiangyin, China; ^2^ Department of Orthopaedics, Tongren Hospital, Shanghai Jiao Tong University School of Medicine, Shanghai, China; ^3^ Department of Emergency, 900 Hospital of The Joint Logistics Team, Dongfang Hospital, Xiamen University, Fuzong Clinical College of Fujian Medical University, Fuzhou, China; ^4^ Clinical Research & Lab Center, Affiliated Kunshan Hospital of Jiangsu University, Kunshan, China

**Keywords:** osteosarcoma, AMPK, PF-06409577, signaling, cell growth

## Abstract

Osteosarcoma (OS) is a common primary bone malignancy. We here investigated the potential activity of PF-06409577, a novel, potent, and direct activator of AMP-activated protein kinase (AMPK), against human OS cells. In established (U2OS, MG-63, and SaOs-2 lines) and primary human OS cells, PF-06409577 inhibited cell viability and proliferation, while inducing cell apoptosis and cell cycle arrest. PF-06409577 induced AMPK activation, mTORC1 inhibition, autophagy induction, and downregulation of multiple receptor tyrosine kinase inOS cells. AMPK inactivation by AMPK*α*1 shRNA, CRISPR/Cas9 knockout, or dominant negative mutation (T172A) was able to abolish PF-06409577-induced activity in OS cells. *In vivo*, PF-06409577 oral administration at well-tolerated doses potently inhibited growth of U2OS cells and primary human OS cells in severe combined immunodeficient mice. AMPK activation, mTORC1 inhibition, autophagy induction, as well as RTK degradation and apoptosis activation were detected in PF-06409577-treated xenografts. In conclusion, activation of AMPK by PF-06409577 inhibits OS cell growth.

## Introduction

Osteosarcoma (OS) is a common primary and malignant bone tumor detected mostly in children, adolescents, and young adults (1). Current clinical therapies, including systemic chemotherapy and local control surgery, have improved OS survival to 70% since the 1970s (1). The metastatic, recurrent, or advanced OS are, however, intrinsically resistant to almost all chemotherapeutic drugs (2, 3). Nowadays, molecularly targeted therapy is the research focus of OS (3), and our group has been testing novel agents against OS cells (4–7).

AMP-activated protein kinase (AMPK) is an evolutionary conserved and ubiquitously expressed serine/threonine protein kinase that is composed of *α*-, *β*-, and *γ*-subunits (8, 9). Phosphorylation of AMPK*α*1-subunit at Thr-172 residue is crucial for its activation (8, 9). AMPK coordinates metabolic pathways in response to energy supply and demand (8, 9). AMPK activation can inhibit human cancer cells *via* different mechanisms (10), including p53 activation, mammalian target of rapamycin (mTOR) complex 1 (mTORC1) inhibition, autophagy induction, and degradation of oncogenes (11, 12). Furthermore, AMPK negatively regulates aerobic glycolysis and cellular biosynthesis to inhibit human cancer cell growth and proliferation (13). AMPK*α*1 expression and Thr-172 phosphorylation are downregulated in many human cancers (14–16). Conversely, overexpression of AMPK*α*1 can suppress human cancer cell growth and proliferation (17). Further supporting the tumor suppressor function for AMPK, a number of anti-cancer agents were found to activate AMPK signaling to induce cancer cell death and apoptosis (18–21).

Existing studies have confirmed that forced activation of AMPK cascade can efficiently inhibit human OS cell growth (22–25). Morishita et al. demonstrated that AMPK activation by AICAR provoked mitochondrial apoptosis in human OS cells (22). Kamel et al. discovered that simvastatin activated AMPK-p38 cascade to inhibit human OS cell growth (23). Fan et al. revealed that 6-gingerol, by activating AMPK cascade, induced proliferation inhibition and apoptosis in OS cells (24). Furthermore, capsaicin-induced OS cell inhibitory activity is associated with AMPK signaling activation (25). We have previously shown that AMPK activation by microRNA-135b inhibited human OS cell growth and proliferation (6). These results indicated that activating AMPK signaling can induce profound OS cell inhibitory activity.

Using traditional AMPK activators (AICAR and metformin, *etc*) in treating cancer remains unlikely because of their off-target toxicities, poor bioavailability, and often low efficiency (26–28). Recent studies have developed a small molecule 6-Chloro-5-[4-(1-hydroxycyclobutyl)phenyl]-1H-indole-3-carboxylic acid (PF-06409577) as a selective, potent, and orally bioactive AMPK activator (29). Unlike other AMPK activators, PF-06409577 is a direct activator of AMPK (29). It directly binds to AMPK subunits, causing robust and sustained AMPK activation (29). In this study, we show that the activation of AMPK by PF-06409577 inhibited human OS cell growth *in vitro* and *in vivo*.

## Material and Methods

### Ethics

All the methods applied in this study were carried out according to the ethics guidelines by all authors’ institutions.

### Reagents and Chemicals

PF-06409577 was purchased from MedKoo Bioscience (Shanghai, China). Antibodies were purchased from Cell Signaling Technologies (Beverly, MA, USA). AICAR, metformin, puromycin, and agents for cell culture were purchased from Sigma-Aldrich (St Louis, MO, USA).

### Culture of Established Human Cell Lines

The established human OS cell lines, U2OS, SaOs-2, and MG-63, as well as the murine bone marrow-derived OB-6 cells were cultured as reported (4, 5, 7). Authentication by STR profiling, population doubling time, and morphology were routinely confirmed as well to verify the genotype. Written-informed consent was obtained from OS patients. The protocols of this study were approved by Ethics Committee of Southeast University according to the Declaration of Helsinki.

### Culture of Primary Human Cells

Surgery-obtained human OS tissues were washed and digested as described (7). Blood vessel cells, immune cells, and fibroblasts were abandoned. The tissue specimens were collected from two OS patients who provided written-informed consent: Patient-1, AJCC:T2N0M0G2, Enneking IIB, male, 12 years old; Patient-2, T1N0M0G2, Enneking IIA, male, 15 years old. The primary human OS cells, Pri_OS-1 and Pri_OS-2, were cultured in the medium described previously (7). The primary human osteoblasts were provided by Dr. Wang (30), which were cultured in *α*-MEM with 10% FBS, and plated at 2 × 10^4^cells/cm^2^. The primary human cells at passages 3–8 were utilized for *in vitro* experiments. The protocols for using primary human cells were approved by the Ethics Board of Southeast University of China according to the principles of Declaration of Helsinki.

### Cell Viability

Cells were seeded onto the 96-well tissue culture plates (5 × 10^3^ cells per well). After the treatment application, cell viability was tested by the routine MTT assay using the described method (4, 5). MTT optical density (OD) at 550 nm was recorded.

### Trypan Blue Staining

Cells were seeded onto the 24-well tissue culture plates (3 × 10^4^ cells per well). Following treatment application, dead cells were positively stained with trypan blue (Sigma, St. Louis, Mo.), and the percentage (%) of trypan blue cells was recorded.

### BrdU assay

BrdU incorporation was tested using a commercial available enzyme-linked immunosorbent assay (ELISA) kit (Cell Signaling Tech) (31). Cells were initially seeded onto the 24-well tissue culture plates (3 × 10^4^ cells per well). Following treatment application, cells were incubated with BrdU (10 μM) for additional 12 h. BrdU OD value at 450 nm was measured.

### Clonogenicity Assay

U2OS cells (5 × 10^4^ cells per treatment) were resuspended in 1 ml of DMEM with 1% agar (Sigma), 10% FBS, and with indicated PF-06409577 treatment. The cell suspension was then added on top of a pre-solidified 1% agar in a 100 mm culture dish. The medium was renewed every 2 days for a total of 10-day period. The number of colonies was then stained and counted.

### Western Blotting

Cells were lysed by the described cell lysis buffer (4, 5). Total cell lysates (30 μg of each treatment per lane) were separated by 8–10% SDS-PAGE gels, and protein samples were transferred onto polyvinylidene fluoride (PVDF) membranes (Millipore, Invitrogen, Shanghai, China). Afterwards, the membranes were blocked, followed by incubation with the indicated primary and secondary antibodies. Detection was accomplished by chemiluminescence with ECL (GE Healthcare, Shanghai, China). Quantification of bands by the ImageJ software was performed as described (4, 5).

### Annexin V FACS

Cells were initially seeded onto the six-well tissue culture plates (2 × 10^5^ cells per well). The detailed protocol of Annexin V FACS assay of cell apoptosis was described previously (32). Annexin V positive cells were gated by FACS as apoptotic cells. Annexin V ratio was recorded.

### TUNEL Staining

Cells were initially seeded onto the 12-well tissue culture plates (5 × 10^4^ cells per well). Following the treatment application, TUNEL (Terminal Deoxynucleotidyl Transferase dUTP Nick End Labeling) *In Situ *Cell Apoptosis Detection Kit (Roche) was utilized to detect nuclear TUNEL staining. TUNEL ratio was recorded from at least 1,000 cell nuclei in five random views per treatment (4).

### Caspase-3/-9 Activity Assay

Cytosolic proteins from 1 × 10^6^ cells per treatment were extracted (4, 5). Twenty micrograms of cytosolic extracts was added to the described caspase assay buffer (4, 5) along with the caspase-3 substrate Ac-DEVD-AFC or the caspase-9 substrate Ac-LEHD-AFC. The released AFC was measured using a spectrofluorometer at 450 nm (Thermo-Labsystems, Helsinki, Finland).

### Nuclear EdU Staining

U2OS cells were initially seeded into six-well plates (at 8 × 10^4^ cells per well), following the treatment applications and cultured for 48 h; an EdU (5-ethynyl-20-deoxyuridine) Apollo-567 Kit (RiboBio, Guangzhou, China) was utilized to quantify cell proliferation. EdU and DAPI dyes were added to the U2OS cells and visualized under a fluorescent microscope. Nuclear EdU ratio was calculated from 800 nuclei in five random views (1 × 200) in each condition.

### Histone/DNA ELISA Assay of Cell Apoptosis

Following the treatment application, the Cell Apoptosis Detection ELISA Kit (Roche, Shanghai, China) was utilized to quantify cell apoptosis according to the protocol. The detailed procedures were described in our previous studies (4, 5). The histone DNA ELISA OD at 405 nm was recorded.

### JC-1 Assaying of Mitochondrial Depolarization

U2OS cells were seeded into six-well plates (8 × 10^3^ cells per well). Following the treatment application of PF-06409577, the JC-1 (Sigma, St Louis, Mo, USA) assay was carried out to test mitochondrial depolarization (“ΔΨ”) *via* a described protocol (33). JC-1 fluorescence intensity was detected at the wavelength of 550 nm, which reflected mitochondrial depolarization intensity. The representative JC-1 images of U2OS cells, integrating both green (550 nm) and red (425 nm) fluorescence images, were presented as well.

### Cell Cycle Analyses

Cells were initially seeded onto the six-well tissue culture plates (1 × 10 5 cells per well). Following the applied treatment, cell cycle distribution was tested by propidium iodide (PI) FACS assay. The detailed protocol was described previously (4). The ratios of G0/1-, S- and G2/M-phases were recorded.

### AMPK Activity Assay

For each treatment, 500 μg total cell lysates were immunoprecipitated with anti-pan-AMPK*α*1 antibody. Within the kinase assay buffer (34), the AMPK activity was measured by adding the AMP-[*γ*-^32^P] ATP mixture and the SAMS (HMRSAMSGLHLVKRR) peptide (34). The phosphocellulose paper (P81) was thereafter added to stop the reaction. After extensive washing, AMPK*α*1 radioactivity was measured using the scintillation counter.

### AMPK*α*1 shRNA

Two different lentiviral AMPK*α*1 shRNAs against non-overlapping sequences of AMPK*α*1 (“S1” and “S2”) were provided by Dr. Sun (18). Cells were initially seeded onto six-well tissue culture plates (1 × 10^5^ cells per well). The lentiviral AMPK*α*1 shRNA (10 μl/ml medium) was added directly to U2OS cells for 24 h. Afterwards, puromycin (1 μg/ml, Sigma) was added for another 96 h. AMPK*α*1 knockdown in the stable cells was verified by Western blotting.

### AMPK*α*1 Knockout by CRISPR/Cas9

The AMPK*α*1 small guide RNA was inserted into the lenti-CRISPR plasmid (Addgene, Shanghai, China) to establish the CRISPR/Cas9-AMPK*α*1-knockout construct. U2OS cells were initially seeded onto the six-well tissue culture plates (1 × 10^5^ cells per well). The construct, provided by Dr. Zhao (35), was transfected to U2OS cells. Two lines of AMPK*α*1-knockout stable cells were selected by puromycin (1.0 μg/ml). AMPK*α*1 knockout was confirmed by Western blotting.

### Light Chain 3B Immunochemistry Staining

As described previously (36, 37), cells were fixed and incubated with anti-LC3B antibody (RFP-conjugated, Genepharm, Shanghai, China). LC3B GFP fluorescence was visualized under a Leica microscope. LC3B RFP puncta formation was detected in autophagic cells.

### AMPK*α*1 Dominant Negative Mutation

As previously described (6), the dominant negative mutant AMPK*α*1 (dn-AMPK*α*1, T172A) pSuper-puro construct was provided by Dr. Lu (12). The dn-AMPK*α*1 construct (0.2 μg/ml medium) was transfected to U2OS cells by Lipofectamine 2000, and stable cells were selected *via* puromycin (1 μg/ml, Sigma). Expression of the exogenous dn-AMPK*α*1 (Flag-tagged) was confirmed by the Western blotting assay.

### Forced AMPK Activation

A constitutively active mutant of AMPK*α*1 (T172D, ca-AMPK*α*1-puromycin) and the empty vector (“Vec”) were received from Dr. Wang at Soochow University (Suzhou, China) (38). U2OS cells were cultured in basic medium (at 60–70% confluence) and transfected with ca-AMPK*α*1 (0.25 μg/ml) by Lipofectamine 2000. To select stable cells, puromycin was added. Expression of exogenous ca-AMPK*α*1 in the stable cells was verified by Western blotting.

### Quantitative Real Time-PCR

Total RNA was extracted by TRIzol reagents and was reverse transcripted (33). qPCR assays were carried out using an ABI Prism 7500 system through the SYBR GREEN PCR Master Mix (Applied Biosystems). *Glyceraldehyde-3-phosphatedehydrogenase* (*GAPDH)* was tested as the reference gene and the internal control. The 2^−ΔΔ^ *^C^* ^t^ method was utilized for data quantification. mRNA primers were provided by Dr. Chen (12).

### *In Vivo* Xenograft Studies

Using a previously described protocol (4), CB.17 severe combined immunodeficient (SCID) male mice (5–6 weeks old, 6 weeks) from the animal facility of Medical School of Southeast University of China (Nanjing, China) were subcutaneously (*s.c.*) injected with U2OS cells or the primary human OS cells in the right flanks at 3 × 10^6^ per mouse. When xenografts were established at about 100 mm^3^ in volume, the SCID mice (10 mice per group) were orally administrated PF-06409577 (10/30 mg/kg in saline) or the vehicle control daily for 24 days. The diameter of xenografted tumors was measured. Tumor volumes (mm^3^) and mice body weights (g) were recorded as described (4, 5). The animal protocols in this study were in accordance with IACUC of Southeast University of China.

### Statistical Studies

*In vitro* experiments were repeated at least three times. The data presented were mean ± standard deviation (SD). Statistical differences were analyzed by one-way ANOVA followed by multiple comparisons performed with *post-hoc* Bonferroni test (SPSS version 20.0, SPSS Co., Chicago, CA). When comparing significances between two treatment groups, a two-tailed unpaired T-test was applied (Excel 2007, Microsoft). Values of *p <*0.05 were considered statistically significant. All the protocols of this study were approved by the Ethics Committee of Southeast University.

## Results

### PF-06409577 Inhibits Human OS Cell Viability and Proliferation

**Figure 1:**
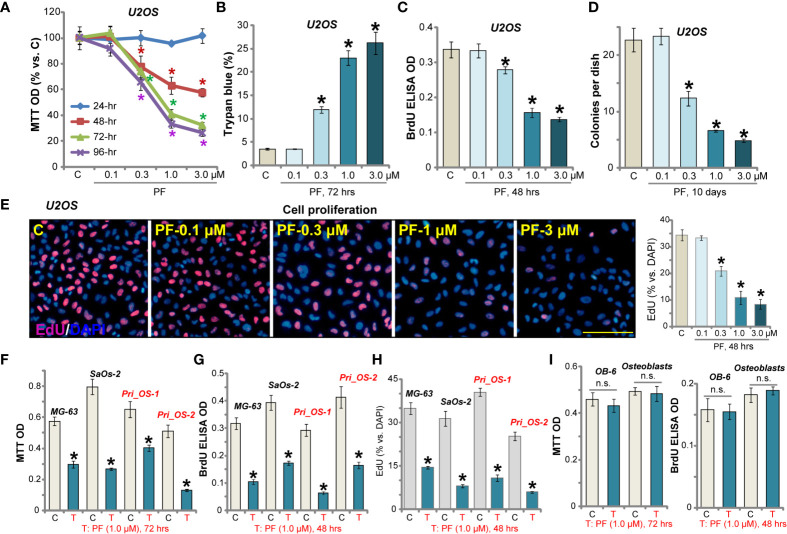
PF-06409577 inhibits human OS cell viability and proliferation. U2OS cells **(A–E)**, MG-63 cells, SaOs-2 cells **(F–H)**, primary human OS cells (“Pri_OS-1/-2”) **(F–H)**, OB-6 cells (“OB-6”) **(I)**, or primary human osteoblasts (“Osteoblasts”) **(I)** were treated with applied concentration of PF-06409577 (“PF”) for indicated time periods. Cell viability, death, and cell proliferation were analyzed by the methods mentioned in the text. “C” stands for cells left untreated for indicated time (same for all figures). For each assay, n = 5 (five replicate wells/dishes). Data were presented as mean ± standard deviation (SD). *p < 0.05 *vs.* “C” cells. “n.s.” stands for non-statistical difference (same for all figures). Experiments were repeated three times, and similar results were obtained each time. Scale bar = 100 μm **(E)**.

The potential effect of PF-06409577 on cultured human OS cells was tested. By performing MTT assay, we show that PF-06409577 efficiently inhibited U2OS cell viability (**Figure 1A**). The AMPK activator dose- and time-dependently decreased MTT OD in U2OS cells (**Figure 1A**). It required at least 48 h to exert a significant anti-survival effect (**Figure 1A**). Further, PF-06409577 dose-dependently increased the number of trypan blue positive (“dead”) U2OS cells (**Figure 1B**). The BrdU incorporation assay and the clonogenicity assay were preformed to test cell proliferation. As shown, in U2OS cells, PF-06409577 dose-dependently decreased BrdU incorporation (in ELISA OD, **Figure 1C**) and the number of colonies (**Figure 1D**). PF-06409577, at 0.3–3 μM, inhibited EdU-positive cell nuclei ratio, further confirming its anti-proliferative activity (**Figure 1E**). At the lowest concentration (0.1 μM), PF-06409577 failed to significantly affect U2OS cell viability and proliferation (**Figures 1A–E**).

Other human OS cells were tested next. Results show that PF-06409577 (1 μM, 48/72 h) significantly inhibited viability (MTT OD) and BrdU incorporation in two other established OS cell lines (MG-63 and SaOs-2, **Figures 1F, G** **)**. In the primary human OS cells (“Pri_OS-1/-2”, derived from different patients), the viability and BrdU incorporation were inhibited as well by the AMPK activator (**Figures 1F, G** **)**. Further studies confirmed that the AMPK activator potently suppressed EdU incorporation in OS cells (results quantified in **Figure 1H**). However, PF-06409577 treatment failed to affect the viability (MTT OD) and proliferation (BrdU incorporation) of murine OB-6 cells and primary human osteoblasts (**Figure 1I**). Together, PF-06409577 potently inhibited human OS cell viability and proliferation.

### PF-06409577 Induces Apoptosis Activation and Cell Cycle Arrest in Human OS Cells

**Figure 2:**
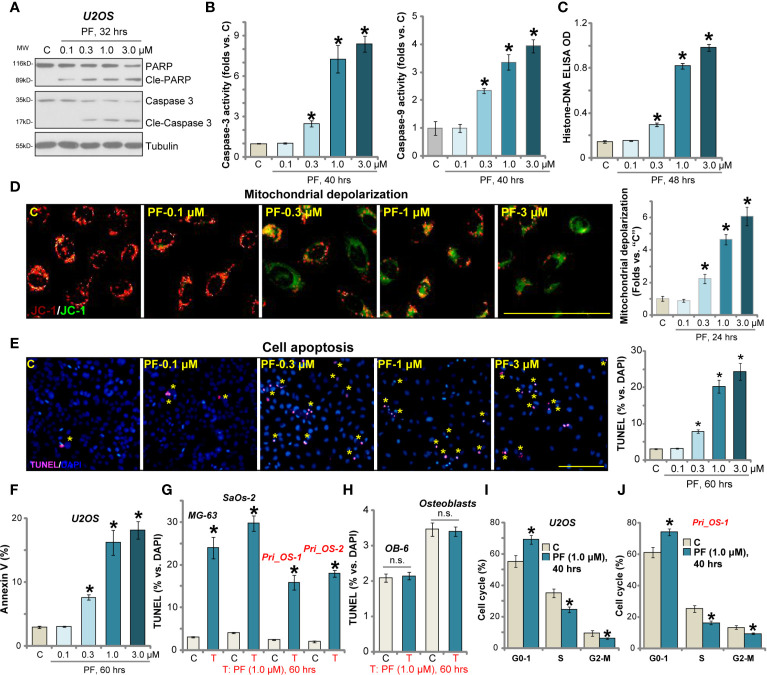
PF-06409577 induces apoptosis activation and cell cycle arrest in human OS cells. U2OS cells **(A–F)**, MG-63 cells, SaOs-2 cells **(G)**, primary human OS cells (“Pri_OS-1/-2”) **(G)**, OB-6 cells (“OB-6”) **(H)**, or primary human osteoblasts (“Osteoblasts”) **(H)** were treated with applied concentration of PF-06409577 (“PF”) for indicated time periods, caspases activation and cell apoptosis were tested by the assays mentioned in the text. U2OS cells **(I)** or the primary human OS cells (“Pri_OS-1”) **(J)** were treated with PF-06409577 (“PF”, 1 μM, 40 h), cell cycle distribution was tested by PI-FACS, and results were quantified. For each assay, n = 5 (five replicate wells/dishes). Data were presented as mean ± standard deviation (SD). *p < 0.05 *vs.* “C” cells. “n.s.” stands for non-statistical difference. Experiments were repeated three times, and each time similar results were obtained. Scale bar = 100 μm **(D, E)**.

The potential effect of PF-06409577 on OS cell apoptosis was examined next. We showed that PF-06409577 dose-dependently induced cleavages of caspase-3 and poly(ADP-ribose) polymerase (PARP) in U2OS cells (**Figure 2A**). Caspase-3 and caspase-9 activities (**Figure 2B**), as well as histone-bound DNA contents (indicating DNA breaks, **Figure 2C**), were increased in PF-06409577 (0.3–3.0 μM)-treated U2OS cells. JC-1 dye assay results in **Figure 2D** demonstrated that PF-06409577 dose-dependently induced mitochondrial depolarization (reflected by JC-1 green monomers accumulation), as the latter is a characteristic marker of mitochondrial apoptosis cascade activation (39). Furthermore, PF-06409577, in a dose-dependent manner, increased TUNEL-positive nuclei (labeled with yellow star, same for all figures) ratio in U2OS cells (**Figure 2E**). Additionally, PF-06409577 (0.3–3.0 μM) significantly increased the number of U2OS cells with Annexin V positive staining (see quantified results in **Figure 2F**). These results confirmed that PF-06409577 provoked apoptosis activation in U2OS cells. At the lowest concentration (0.1 μM), PF-06409577 failed to induce significant apoptosis activation (**Figures 2A–F**).

In MG-63 cells and SaOs-2 cells, PF-06409577 treatment (1 μM, 60 h) induced apoptosis activation, evidenced by TUNEL-positive nuclei ratio increase (**Figure 2G**). Similarly, significant apoptosis activation was detected in the primary human OS cells (Pri_OS-1/Pri_OS-2) with PF-06409577 stimulation (**Figure 2G**). Yet in OB-6 cells and the primary human osteoblasts, PF-06409577 stimulation failed to induce significant apoptosis activation (TUNEL assay, **Figure 2H**).

Cell cycle progression is essential for cancer cell proliferation and survival. Through employing a propidium iodide (PI)-FACS assay, we show that PF-06409577 (1 μM, 40 h) disrupted cell cycle progression in U2OS cells and caused increased G0/1-phase cells but decreased S-phase cells (results quantified in **Figure 2I**). Similarly, G1-S arrest was detected in PF-06409577-treated primary human OS cells (“Pri_OS-1”) (**Figure 2J**).

### PF-06409577 Activates AMPK Signaling Cascade in Human OS Cells

**Figure 3:**
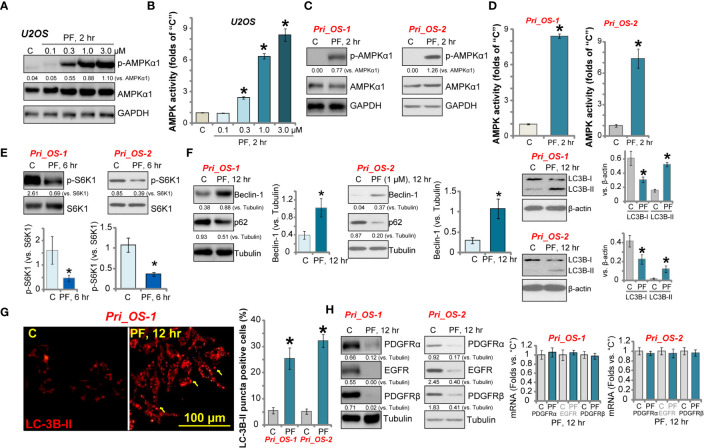
PF-06409577 activates AMPK signaling cascade in human OS cells. U2OS cells **(A, B)** or the primary human OS cells (“Pri_OS-1” and “Pri_OS-2”, **C**–**H**) were treated with applied concentration of PF-06409577 for indicated time; expression of listed proteins in total cell lysates was tested by Western blotting assay **(A**, **C**, **E**, **F**, **H)**; relative AMPK activity was also tested **(B, D)**. LC3B-RFP (red fluorescence protein) puncta formation was shown, LC3B-RFP-puncta positive cell ratio was quantified (from 500 cells in each condition, **G**). Expression of *PDGFRα/β* and *EGFR* mRNA was tested by qPCR assays **(H)**. Blot data were quantified and normalized to each loading control. For each assay, n = 5 (five replicate welsl/dishes) **(B, D)**. Data were presented as mean ± standard deviation (SD) **(B, D)**. *p < 0.05 *vs.* “C” cells **(B, D)**. Experiments were repeated three times, and each time similar results were obtained. Scale bar = 100 μm **(G)**.

PF-06409577 is a novel and direct AMPK activator (29, 40). Western blotting assay results in **Figure 3A** show that PF-06409577 dose-dependently increased the phosphorylation of AMPK*α*1 (at Thr-172) in U2OS cells. AMPK activity was significantly increased in PF-06409577 (0.3–3.0 μM)-treated U2OS cells (**Figure 3B**). In primary human OS cells (“Pri_OS-1” and “Pri_OS-2”), PF-06409577 (1 μM) significantly increased AMPK*α*1 phosphorylation (**Figure 3C**) and AMPK activity (**Figure 3D**), which indicated AMPK activation.

Activated AMPK inhibits human cancer cells *via* various mechanisms, including mTORC1 inhibition (41–43), autophagy induction (43–45), and oncogene degradation (11, 12). We found that PF-06409577 (1 μM) inhibited the phosphorylation of S6K1 (at Thr-389) in Pri_OS-1 and Pri_OS-2 cells (**Figure 3E**), indicating the inhibition of mTORC1 (46, 47). Further studies show that PF-06409577 (1 μM) induced autophagy-like changes, causing the upregulation of Beclin-1, the degradation of p62, and importantly LC3B-I to LC3B-II conversion in the primary OS cells (**Figure 3F**). Autophagy activation was further confirmed by the formation of LC3B-RFP (red fluorescence protein) puncta in PF-06409577-treated Pri_OS-1 and Pri_OS-2 cells (**Figure 3G**). The yellow arrow indicated characteristicLC3B-RFP fluorescence puncta (**Figure 3G**).

Chen et al. have shown that activation of AMPK can induce lysosomal translocation and degradation of multiple receptor tyrosine kinases (RTKs), including PDGFR*α*, PDGFR*β*, and EGFR (12). In line with that findings, PDGFR*α*/*β* and EGFR protein expression levels were significantly decreased in PF-06409577 (1 μM)-treated Pri_OS-1 and Pri_OS-2 cells (**Figure 3H**). qPCR assay results confirmed that mRNA expressions of PDGFR*α*/*β* and EGFR were unchanged (**Figure 3H**). Together, PF-06409577 induced AMPK activation, mTORC1 inhibition, autophagy activation, and RTK degradation in OS cells.

### AMPK Activation Mediates PF-06409577-Induced Activity in OS Cells

**Figure 4:**
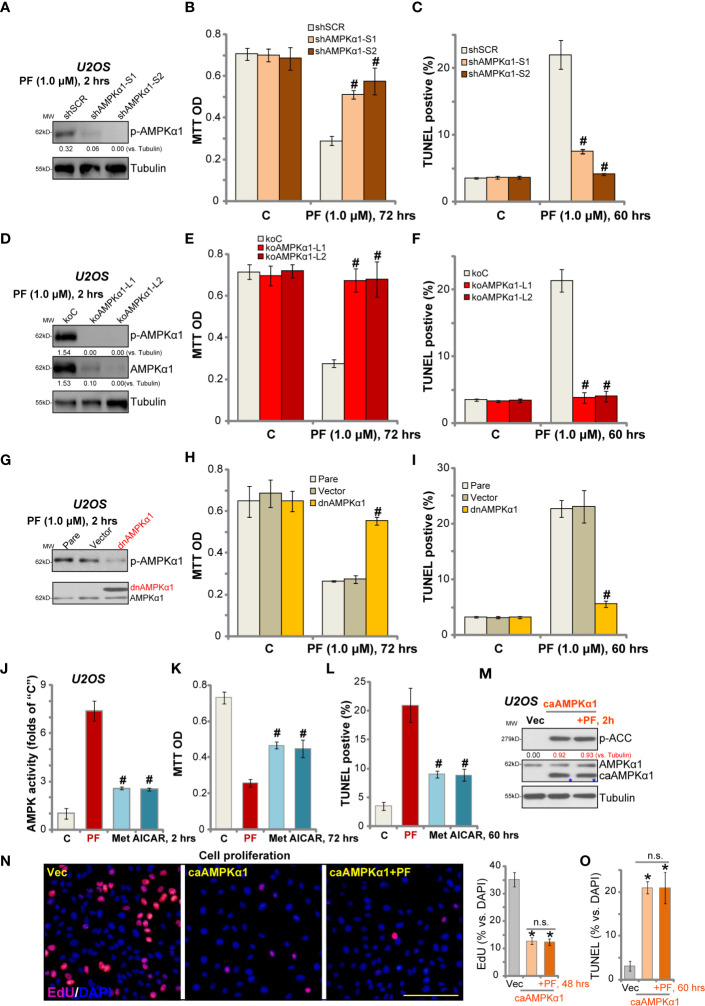
AMPK activation mediates PF-06409577-induced activity in OS cells. Stable U2OS cells, with AMPK*α*1 shRNA (“shAMPK*α*1-S1/-2”) or scramble control shRNA (“shSCR”), the CRISPR/Cas9-AMPK*α*1-knockout construct (“koAMPKα1-L1/L2”) or the control CRISPR/Cas9 construct (“koC”), as well as the dominant negative AMPK*α* (T172A, “dnAMPK*α*1”) or the empty vector (pSuper-puro, “Vector”), were treated with PF-06409577 (“PF”, 1 μM) for indicated time, expressions of listed proteins in total cell lysates were tested by Western blotting assay **(A**, **D**, **G)**; Cell viability and apoptosis were tested by MTT assay **(B**, **E**, **H)** and TUNEL staining assay **(C**, **F**, **I)**, respectively. U2OS cells were treated with PF-06409577 (“PF”, 1 μM), AICAR (10 mM) and metformin (“Met”, 5 mM) for applied time periods, the relative AMPK activity was shown **(J)**; Cell viability and apoptosis were tested by MTT assay **(K)** and TUNEL staining assay **(L)**, respectively. Stable U2OS cells-bearing the constitutive-active AMPKα1 (“caAMPKα1”, T172D) **(M–O)** were further treated with or without PF-06409577 (“PF”, 1 μM), control cells were treated with the empty vector (“Vec”) **(M–O)**. Cells were further cultured for applied time periods, expressions of listed proteins were shown **(M)**; Cell proliferation (EdU ratio, **N**), and apoptosis (TUNEL-positive nuclei ratio, **O**) were tested. “Pare” stands for the parental control cells **(G–I)**. Blot data was quantified and normalized to each loading control. Data were presented as mean ± standard deviation (SD). ^#^ *p* < 0.05 *vs.* control cells with PF-06409577 treatment **(A–I)**. ^#^ *p* < 0.05 *vs.* PF-06409577 treatment **(J–L)**. *p < 0.05 *vs.* “Vec” cells **(N, O)**. “n.s.” stands for non-statistical difference. Experiments were repeated three times, and each time similar results were obtained. Scale bar = 100 μm **(N)**.

Next shRNA strategy was applied to knockdown AMPK*α*1 in OS cells. Two sets of lentivirus with non-overlapping sequences of AMPK*α*1 shRNA [“-S1/S2”, from Dr. Sun (18)] were added to U2OS cells. With puromycin selection, the stable cells were established. Western blotting results in **Figure 4A** confirmed that PF-06409577-induced AMPK activation was significantly inhibited by the lentiviral shRNA. Significantly, as compared to control cells with scramble control shRNA (“shSCR”), PF-06409577-induced viability reduction (**Figure 4B**) and apoptosis activation (TUNEL ratio increase, **Figure 4C**) were dramatically attenuated in AMPK*α*1-silenced U2OS cells.

Further, CRISPR/Cas9 gene editing method was utilized to completely knockout AMPK*α*1 [using the construct provided by Dr. Zhao (35)]. AMPK*α*1 was depleted in the two lines of stable U2OS cells with CRISPR/Cas9-AMPK*α*1-knockout construct (“koAMPK*α*1-L1/L2”) (**Figure 4D**). PF-06409577-induced AMPK*α*1 phosphorylation was blocked in AMPK*α*1-knockout cells (**Figure 4D**). Consequently, PF-06409577-induced viability reduction (**Figure 4E**) and apoptosis (**Figure 4F**) were completely blocked by AMPK*α*1 knockout.

Next, a dominant negative mutant AMPK*α*1 (“dnAMPK*α*1”, T172A) was transfected to U2OS cells, and stable cells were established. Western blotting results show that dnAMPK*α*1 (Flag-tagged) largely inhibited PF-06409577-induced AMPK*α*1 phosphorylation (**Figure 4G**). PF-06409577-induced cytotoxicity in USO2 cells was significantly alleviated by dnAMPK*α*1 (**Figures 4H, I** **)**. This genetic evidence together suggested that the activation of AMPK mediated PF-06409577-induced cytotoxicity in USO2 cells.

Importantly, in U2OS cells, PF-06409577 (1 μM)-induced AMPK activation was significantly more potent than that by known AMPK activators AICAR (10 mM) and metformin (5 mM) (**Figure 4J**) at much higher concentrations (**Figure 4J**). Consequently, PF-06409577-induced cytotoxicity (MTT OD reduction, **Figure 4K**) and apoptosis activation (TUNEL ratio increase, **Figure 4L**) in U2OS cells were more significant than AICAR and metformin.

We hypothesized that the activity of PF-06409577 should be compromised in AMPK pre-activated OS cells. A constitutive-active AMPK*α*1 (“caAMPK*α*1”, T172D, from Dr. Wang (38) was transduced to U2OS cells, and stable cells were established. As shown, p-AMPK*α*1 levels were significantly increased in caAMPK*α*1-expresssing (“blue star” labeled) U2OS cells (**Figure 4M**). Similar to the action by PF-06409577, forced activation of AMPK by caAMPK*α*1 produced OS cell inhibitory activity, as it inhibited cell proliferation (EdU incorporation, **Figure 4N**) and induced apoptosis activation (TUNEL-positive nuclei ratio increase, **Figure 4O**). Importantly, in caAMPK*α*1-bearing U2OS cells, PF-06409577 failed to further increase AMPK*α*1 phosphorylation (**Figure 4M**), nor did it further inhibit OS cells (**Figures 4N, O** **)**.

### PF-06409577 Oral Administration Inhibits U2OS Xenograft Tumor Growth in SCID Mice

**Figure 5:**
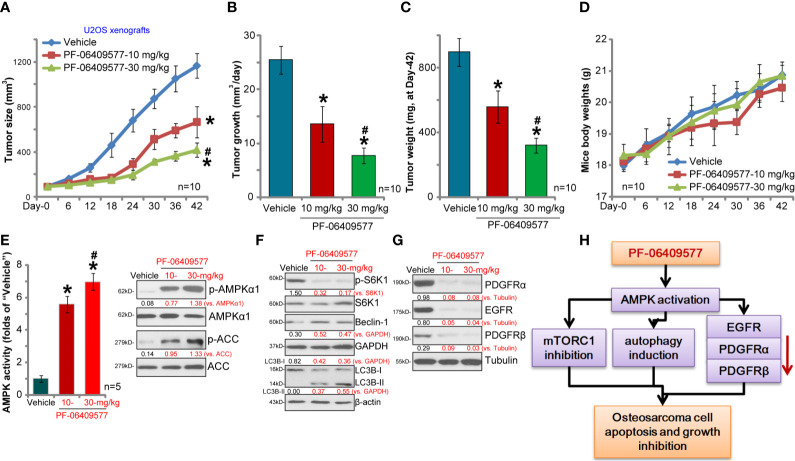
PF-06409577 oral administration inhibits U2OS xenograft tumor growth in SCID mice. The U2OS xenograft tumor-bearing SCID mice (10 mice per group) were administrated with vehicle control (“Vehicle”, saline) or PF-06409577 (10/30 mg/kg, oral gavage, daily for 24 days), tumor volumes **(A)** and mice body weights **(D)** were measured at every 6 days for a total of 42 days. The estimated daily tumor growth, in mm^3^ per day, was calculated as described **(B)**. At Day-42, tumors of all three groups were weighted individually **(C)**. At Day-6, one xenograft USO2 tumor per group was recovered, after which each tumor was cut into five random pieces (n = 5), and expression of the listed proteins was assessed by Western blotting analyses **(F, G)**, with relative AMPK activity tested as well **(E)**. The proposed signaling cascade of this study is shown **(H)**. Blot data were quantified and normalized to each loading control. Data were presented as mean ± standard deviation (SD). *p < 0.05 *vs.* “Vehicle” **(A–C**, **F)**. ^#^*p* < 0.05 *vs.* PF-06409577 at 10 mg/kg **(A–C**, **E)**.

We tested the activity of PF-06409577 *in vivo*. As described (7), U2OS cells were injected *s.c.* to the flanks of SCID mice. Within 2–3 weeks, the volume of each tumor was close to 100 mm^3^ (“Day-0”); mice were then randomly assigned into three groups. Ten mice of each group were orally administrated with either PF-06409577 (10/30 mg/kg, daily for 24 days) or vehicle control. The tumor growth curve results show that PF-06409577 oral administration significantly inhibited U2OS tumor growth in SCID mice (**Figure 5A**). When analyzing daily tumor growth, which was calculated by (tumor volume at Day-42 subtracting tumor volume at Day-0)/42, we found that PF-06409577-treated U2OS tumors grew significantly slower than control tumors (**Figure 5B**). All tumors were isolated at Day-42 and weighted. Results show that PF-06409577-treated tumors weighted lower than control tumors (**Figure 5C**). PF-06409577 at 30 mg/kg was more potent than at 10 mg/kg in inhibiting U2OS tumor growth (**Figures 5A–C**). The mice body weights were not significantly different between the three groups (**Figure 5D**). Thus, PF-06409577 oral administration potently inhibits U2OS xenograft tumor growth in SCID mice.

To assess signaling changes in U2OS tumor tissues, one tumor from each group was isolated (in total three tumors) at Day-6, and signaling proteins in tumor lysates were tested. As demonstrated, PF-06409577 administration activated AMPK signaling in U2OS xenografts and increased AMPK activity (**Figure 5E**). In line with the signaling changes *in vitro*, PF-06409577 treatment *in vivo* induced S6K1 inhibition (**Figure 5F**), Beclin-1 upregulation, and LC3B-I to LC3B-II conversion (**Figure 5F**), as well as PDGFR*α*, PDGFR*β*, and EGFR degradation (**Figure 5G**) in U2OS xenograft tissues. These results suggest that PF-06409577 administration induced AMPK activation, mTORC1 inhibition, autophagy activation and RTK downregulation in U2OS xenograft tumor lysates. Therefore, the *in vivo* findings are consistent with the *in vitro* signaling studies (see the proposed signaling pathway of this study, **Figure 5H**).

### PF-06409577 Administration Inhibits Primary Human OS Cell Growth in SCID Mice

**Figure 6:**
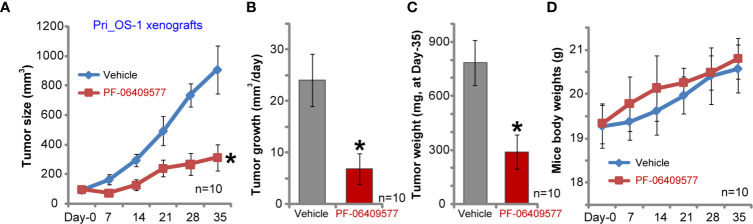
PF-06409577 administration inhibits primary human OS cell growth in SCID mice. SCID mice bearing Pri_OS-1 xenograft tumors were administrated with vehicle control (“Vehicle”, saline) or PF-06409577 (10 mg/kg, oral gavage, daily for 24 days). Tumor volumes **(A)** and mice body weights **(D)** were measured at every 7 days for a total of 35 days. The estimated daily tumor growth, in mm^3^ per day, was calculated **(B)**. At the end of animal experiments, all tumors were weighted individually **(C)**. Data were presented as mean ± standard deviation (SD). *p < 0.05 *vs.* “Vehicle” **(A–C)**.

The primary human OS cells, Pri_OS-1, were *s.c.* injected to the flanks of SCID mice. Within three weeks, the xenografts bearing primary OS cells were established, with a tumor volume close to 100 mm^3^. Following PF-06409577 oral administration (10 mg/kg, daily for 24 days), the growth of primary Pri_OS-1 cells *in vivo* was largely inhibited (**Figure 6A**). By calculating daily tumor growth, we again found that PF-06409577 administration suppressed primary Pri_OS-1 cell growth *in vivo* (24.09 ± 5.10mm^3^ per day *vs.* 6.92 ± 2.91 mm^3^ per day, *p* < 0.05) (**Figure 6B**). At Day-35, Pri_OS-1 tumors were isolated and weighted. Results demonstrated that tumors in PF-06409577 treatment group weighted much lower than the control tumors (786.25 ± 123.86 mg *vs.* 290.72 ± 94.83 mg, *p* < 0.05) (**Figure 6C**). The body weights of mice were not significantly different between the two groups (**Figure 6D**). These results confirmed that PF-06409577 oral administration inhibited primary human OS cell growth *in vivo*.

## Discussion

AMPK is essential in regulating a number of key cellular activities, from energy metabolism, cell mitosis, survival, growth to apoptosis and autophagy (27, 48). It has been proposed that under certain circumstances, AMPK activation could be pro-survival, though it depends on the intensity of the activation. Weak or moderate AMPK activation might promote cell survival. However, sustained or intensified AMPK activation would inhibit cancer cell growth and survival and promote cell apoptosis (26). The activity of AMPK could increase over 100-fold while phosphorylating conserved threonine residue (Thr-172) within the activation loop at *α*1 subunit (8, 27).

In the current study, we show that PF-06409577 potently induced AMPK activation in human OS cells, which led to significant AMPKα1 phosphorylation and AMPK activity enhancement. Significant AMPK activation was also detected in the PF-06409577-treated U2OS tumor lysates. Significantly, PF-06409577 potently inhibited OS cells growth and proliferation, whiling inducing cell apoptosis. Activation of AMPK mediated PF-06409577-induced cytotoxicity against OS cells. AMPK inactivation by AMPK*α*1 silencing, CRISPR/Cas9 knockout, or dominant negative mutation (T172A) abolished the cytotoxicity of PF-06409577 in OS cells. Forced activation of AMPK by caAMPK*α*1 mimicked PF-06409577-induced actions and inhibited OS cell proliferation. Importantly, PF-06409577 failed to further induce OS cell apoptosis with AMPK pre-activation by caAMPK*α*1.

mTOR hyper-activation is detected in human OS. Our previous studies have shown that targeting mTOR by XL388, a novel mTOR kinase inhibitor, potently inhibited human OS cell growth *in vitro* and *in vivo* (7). One primary mechanism of AMPK-mediated anti-cancer activity is to inhibit mTORC1 (41). AMPK, when activated, can phosphorylate and activate TSC2 (tuberous sclerosis protein 2), thereby blocking mTORC1 activation (41). Additionally, activated AMPK directly phosphorylates and in-activates mTORC1 component Raptor (regulatory associated protein of mTOR) to inhibit mTORC1 activation (49). We here show that PF-06409577 significantly inhibited mTORC1 activation (S6K1 phosphorylation) in established and primary human OS cells. In PF-06409577-treated U2OS xenografts, mTORC1 (p-S6K1) blockage was also detected, which could be used to explain its superior activity against OS cells.

Simultaneous activation of several key RTKs in OS cells would lead to persistent activation of downstream oncogenic signaling cascades and promote OS survival, proliferation, and growth (50). Therapies that target only one single RTK have had very limited success (50). Chen et al. have proposed a novel mechanism for AMPK-dependent anti-cancer actions: by degrading multiple RTKs. AMPK activation can promote RTKs (PDGFR*α*, PDGFR*β*, and EGFR) lysosomal translocation, and degradation (12). In line with these findings, we show that PF-06409577 induced downregulation of PDGFR*α*, PDGFR*β*, and EGFR in OS cells. mRNA expressions of these RTKs were however unchanged. Importantly, RTK degradation was also detected in PF-06409577-treated U2OS xenografts. This could also explain the potent activity against OS cells by the AMPK activator.

In human OS cells, PF-06409577 can induce rapid and potent AMPK activation at nM concentrations, which is certainly more efficient than other known AMPK activators. One possibility is that PF-06409577 directly binds to AMPK subunits, causing robust and sustained AMPK activation (29, 40). In OS cells, AMPK activation, cytotoxicity, and apoptosis induced by PF-06409577 were significantly more powerful than the known AMPK activators, including AICAR and metformin. Another benefit of this novel AMPK activator is its oral bio-availability (29, 40), as PF-06409577 oral administration in SCID mice potently inhibited growth of xenograft tumors of U2OS cells and primary OS cells. These results suggest that PF-06409577 could have important therapeutic value for OS.

## Conclusion

We conclude that the activation of AMPK by PF-06409577 potently inhibits OS cell growth *in vitro* and *in vivo*.

## Data Availability Statement

The original contributions presented in the study are included in the article/supplementary material. Further inquiries can be directed to the corresponding authors.

## Ethics Statement

The animal study was reviewed and approved by Institutional Animal Care and Use Committee of Southeast University of China.

## Author Contributions

All the listed authors in the study carried out the experiments, participated in the design of the study and performed the statistical analysis, conceived of the study, and helped to draft the manuscript. All authors contributed to the article and approved the submitted version.

## Funding

This work is supported by the Young medical talents of Jiangsu Province (QNRC2016132). The funders had no role in study design, data collection and analysis, decision to publish, or preparation of the manuscript.

## Conflict of Interest

The authors declare that the research was conducted in the absence of any commercial or financial relationships that could be construed as a potential conflict of interest.
